# Expression and Role of the Intermediate-Conductance Calcium-Activated Potassium Channel KCa3.1 in Glioblastoma

**DOI:** 10.1155/2012/421564

**Published:** 2012-05-17

**Authors:** Luigi Catacuzzeno, Bernard Fioretti, Fabio Franciolini

**Affiliations:** Dipartimento di Biologia Cellulare e Ambientale, Universita' di Perugia, Via Pascoli 1, I-06123 Perugia, Italy

## Abstract

Glioblastomas are characterized by altered expression of several ion channels that have important consequences in cell functions associated with their aggressiveness, such as cell survival, proliferation, and migration. Data on the altered expression and function of the intermediate-conductance calcium-activated K (KCa3.1) channels in glioblastoma cells have only recently become available. This paper aims to (i) illustrate the main structural, biophysical, pharmacological, and modulatory properties of the KCa3.1 channel, (ii) provide a detailed account of data on the expression of this channel in glioblastoma cells, as compared to normal brain tissue, and (iii) critically discuss its major functional roles. Available data suggest that KCa3.1 channels (i) are highly expressed in glioblastoma cells but only scantly in the normal brain parenchima, (ii) play an important role in the control of glioblastoma cell migration. Altogether, these data suggest KCa3.1 channels as potential candidates for a targeted therapy against this tumor.

## 1. Introduction

Glioblastomas are the most common and aggressive among primary brain tumors. In spite of the intensive basic and clinical studies, only minor successes have been witnessed over the last decades. One-third of patients keep surviving no longer than one year from diagnosis, and average life expectancy remains dismal (12–15 months), even when radical surgical resection, chemo- and radiotherapy can be applied. The major problem with glioblastomas is their highly migratory and invasive potential into the normal brain tissue that prevents complete surgical removal of tumor cells and the extreme resistance of these cells to standard treatments [1]. To worsen the outcome of the disease is the presence in the tumor mass of a recently identified subpopulation of highly tumorigenic stem-like glioblastoma cells possessing even more invasive power, chemo- and radio-resistance than nonstem tumor cells, that are also thought to be responsible for the commonly observed tumor relapses [2–4].

Glioblastomas are characterized by a large number and variety of genetic mutations that heavily disregulate the major signaling pathways controlling cell survival, proliferation, differentiation, and invasion [5]. Among the disregulated pathways found in glioblastoma cells there are those controlling the expression of ion channels, transmembrane proteins endowed with a permeation pore that allows the passage of ions. Usually ion channels are selectively permeable to one particular ion and can open and close their permeation pore in response to chemical and physical stimuli, such as neurotransmitters, modulators, and changes in the membrane potential [6]. Ion channels have been found to be involved in several cellular functions, hallmarks of cancer cell aggressiveness, such as proliferation, apoptosis, and migration. In most cases their contribution consists in regulating two important cellular parameters, the cell volume and the intracellular Ca^2+^ concentration ([Ca^2+^]_i_) [7, 8].

By allowing the movement of K and Cl ions through the plasmamembrane, and the osmotically driven water flux, ion channels critically control the changes of cell volume that are functionally relevant for glioblastoma cells. For example, a premitotic volume condensation (PVC) is required for glioblastoma cells to switch from a bipolar into a round cell morphology just prior cell division. Notably, this process requires the opening of Cl-selective ClC-3 channels, that are markedly upregulated in glioblastoma cells as compared to healthy astrocytes [9–12]. Similarly, a cell volume reduction, the so-called apoptotic volume decrease (AVD), was observed during the staurosporine- or TRAIL (TNF-alpha-related apoptosis inducing ligand)-induced apoptosis of glioblastoma cells, and also in this case it was found to be sustained by a Cl channel flux, being prevented by inhibitors of Cl channels [13]. Cell migration and invasion through the narrow extracellular spaces of the brain parenchyma also require major changes in cell volume. These processes in addition to the ClC-3 channels discussed above require the activity of Ca^2+^-activated K-selective BK channels, likewise markedly upregulated in glioblastoma cells as compared to healthy astrocytes [14–16].

The important role of the Ca^2+^ signals in the development of glioblastoma has recently been reviewed [17]. Notably, ion channels play a critical role to this regard; besides sustaining directly the Ca^2+^ influxes (through Ca^2+^-permeable channels) they can influence the entry of extracellular Ca^2+^ ions by modulating the membrane potential that controls the driving force for Ca^2+^ influx. Ca^2+^ influx through the TRPC family of Ca^2+^-permeable channels has indeed been shown to modulate glioblastoma cell cycle progression [18–20] and to induce a CaMKII-dependent activation of ClC-3 during premitotic volume condensation [12]. In addition, glioblastoma cell migration has been shown to be accompanied by intracellular Ca^2+^ oscillations that are instrumental to promote the kinase-dependent detachment of focal adhesions during cell rear retraction [21, 22], and these intracellular Ca^2+^ oscillations can be significantly affected by the membrane hyperpolarization determined by the activity of K channels [23].

Perhaps the best suited ion channels to play a role in tumor development are the Ca^2+^-activated K (KCa) channels, as they are at the cell crossroad where Ca^2+^ influx, membrane potential, and outward ion fluxes, all processes governed by KCa channels, integrate to modulate a large array of cellular processes [24]. KCa channels are subdivided into three major classes according to their single channel conductance: large conductance (150–300 pS) K channels (BK_Ca_ or KCa1), small conductance (2–20 pS) K channels (SK or KCa2.1, KCa2.2, KCa2.3), and intermediate conductance (20–60 pS) K channels (IK_Ca_ or KCa3.1). Each subclass has specific biophysical and pharmacological properties that allow to identify them. KCa1 channels, encoded by the *Kcnma1* gene, are broadly expressed in various tissues. They are regulated by cytoplasmic Ca^2+^ but also by membrane potential. In the absence of Ca^2+^, KCa1 channels can be activated only with extreme (nonphysiological) depolarizations. Elevations in cytoplasmic [Ca^2+^] shift the range of activating voltages to more negative potentials. Near resting potentials, the EC50 of the KCa1 is in the micromolar range. Paxilline, iberiotoxin, and low concentrations of tetraethyl ammonium are potent and specific inhibitors of the KCa1 channel. The KCa2.x channels are voltage independent but more sensitive to Ca^2+^ (EC50 in submicromolar range) due to the presence of calmodulin associated with the C-terminus that works as Ca^2+^ sensor. Apamine, but not paxilline or iberiotoxin, can selectively block the KCa2.x channels. The KCa3.1 channels, like the KCa2.x channels, are voltage independent but gated by intracellular Ca^2+^ that binds to calmodulin and opens the channel. Clotrimazole and its derivative TRAM-34 are potent inhibitors of the KCa3.1 channels, discriminating them from other KCa channels.

KCa3.1 channels are expressed in a variety of normal and tumor cells, where they participate in important cell functions such as cell cycle progression, migration, and epithelial transport, by controlling the cell volume and the driving force for Ca^2+^ influx [25–27]. Here we review the major progresses that have led to our present understanding of the expression and role of the KCa3.1 channels in glioblastoma.

## 2. General Properties of the KCa3.1 Channel

The KCa3.1 channel has the overall architecture of the voltage-gated K (Kv) channel superfamily, with four subunits, each containing six transmembrane domains (S1–S6) and a pore domain (P loop) located between S5 and S6. The S4 domain, which confers voltage sensitivity to the Kv channels, shows in KCa3.1 channels only two positively charged aminoacids, as compared to the 4–7 charged residues of voltage-gated K channels. Channel activation is, therefore, voltage independent. The KCa3.1 channel is gated instead by the binding of intracellular Ca^2+^ to calmodulin, a Ca^2+^-binding protein that is constitutively associated with the C terminus of each channel subunit [28–30]. This Ca^2+^-dependent gating is similar to that displayed by the KCa2.x channel family but distinct from KCa1 channels, where the Ca^2+^-dependent module is intrinsic to the channel *α* subunit [24]. Patch-clamp experiments in several cell types, including glioblastoma, give IC50s for KCa3.1 channel activation by Ca^2+^ of 200–400 nM [31, 32], consistent with those found for the cloned channel [33–35]. The high Ca^2+^ sensitivity of the KCa3.1 channel allows its activation by submicromolar Ca^2+^ levels, easily reached upon Ca^2+^ release from intracellular stores or influx through Ca^2+^ permeable channels. A four-state gating scheme was proposed for KCa3.1 channels, with Ca^2+^-dependent transitions dependent on the [Ca^2+^]_i_ in a nonlinear manner [36]. This peculiarity, not shared by the KCa2.x channel family [37], is related to the channel behaviour at saturating [Ca^2+^]_i_, as elevated divalent concentrations have been reported to block the channel [36, 38]. The most studied KCa3.1 mRNA is the 2.1 kb form, but other transcripts have been reported in humans [34, 35]. Three distinct *Kcnn4* cDNAs that are designated as *Kcnn4a*, *Kcnn4b*, and *Kcnn4c* encoding 425, 424, and 395 aminoacid proteins, respectively, were isolated from the rat colon, and several differences in the functional expression and pharmacological properties of the different isoforms were found [39].

The KCa3.1 channels are target for several inhibitory and activatory agents (for an exhaustive review see [40]). Two structurally distinct groups of KCa3.1 channel blockers, peptidic and nonpeptidic, have been found which also differ for their binding site on the channel protein. Among the peptidic blockers, maurotoxin and charybdotoxin display the strongest potency. Maurotoxin, is a 34-aminoacid toxin cross-linked by four disulfide bridges [41]. Lys23 of the toxin binds to the pore filter of the channel from the extracellular side, and a *π*-*π* interaction between tyr32 of the toxin and a cluster of aromatic residues in the channel pore vestibule stabilizes the interaction [42]. Maurotoxin is not selective for KCa3.1 channels, being also a potent blocker of some members of Kv channels [41]. Charybdotoxin (ChTX), a 37-aminoacid toxin, displays a block mechanism similar to maurotoxin, and poor selectivity, blocking effectively other ion channels including KCa1 channels [43]. Several nonpeptidic molecules have been found to block KCa3.1 channels, such as the vasodilator cetiedil [44, 45], the antimycotic triarylmethane clotrimazole (CTL, [46]), and the antihypertensive L-type Ca^2+^ channel blocker nifedipine [47]. From chemical modification of cetiedil several more potent KCa3.1 channel blockers were obtained. The investigation of one of these compounds, the UCL 1608, suggests that they interact with a lipophilic-binding site located within the membrane [48]. Also the chemical modification of the poorly selective CTL has led to the production of several more effective KCa3.1 channel blockers, including the triarylmethanes TRAM-34 [49] and ICA-17043 [50]. TRAM-34 is so far the best probe to study the roles of KCa3.1 channels, being much more selective than CTL [49]. An excellent work has conclusively delineated the properties of the KCa3.1 channel binding site for TRAM-34 [51]. These authors found that the TRAM-34 analogue and membrane impermeant TRAM-30 blocked the channel only when applied from inside, and the interaction of TRAM-34 with the channel required the P-loop aminoacid Thy250 and the S6 segment aminoacid Val275, both likely facing a large water-filled cavity localized below the narrow selectivity filter of the channel. They thus concluded that the TRAM-34 binding site is accessible from the cytoplasmic side and lays well up inside the inner vestibule. The same work has also found that the dihydropyridines-binding site is likely different from the TRAM-34 binding site, as the same mutation does not alter the blocking action of nifedipine [51]. Starting from nifedipine as lead compound, the 4-phenil-4H-pyrans and the related cyclohexadienes were obtained [52, 53], of which cyclohexadiene 4 represents the most potent blocker of KCa3.1 channel. Particularly interesting for KCa3.1 channel targeting in glioblastomas is the analogue compound bicycle hexadiene lactone 16, that displays a 10-fold enrichment in brain tissue [53].

From the early discovery of 1-ethyl-2-benzimidazolinone (EBIO) as KCa3.1 channel activator [54], much effort has been devoted to increase its potency and selectivity. Potency was initially improved with the introduction of DC-EBIO [55], and more recently with NS309 [56]. Selectivity on the contrary has been more difficult to increase since these compounds activate also KCa2.x channels [40]. The mechanism of action of KCa3.1 channel activators, and the location and structure of their binding sites have been only partially clarified [57, 58]. The potency of all KCa3.1 channel activators depends on Ca^2+^, as they are totally ineffective in its absence [54, 57, 58]. The origin of this Ca^+2^ dependence is still unclear. 

## 3. KCa3.1 Channel Modulation by Intracellular Messengers

### 3.1. Kinase Regulation

Several studies have described a rundown of the KCa3.1 channel activity in ATP-free internal milieu that can be restored after the readdition of ATP [59], suggesting the involvement of kinases in the process. In accordance, several kinases such as PKC, PKA, and PI3Ks have been shown to regulate the KCa3.1 channels [59–61], although not through the direct phosphorylation of the channel *α* subunit [59, 61, 62]. Only the nucleotide diphosphate kinase (NDPK) has been shown to phosphorylate the KCa3.1 channel alpha subunit (at the hist358) [63], and a similar action could be exerted by adenosine monophosphate kinase (AMPK), although the aminoacid residue targeted in this case has not been identified [64]. It is possible that NDPK or AMPK represent integration points for other kinases found to modulate KCa3.1 channels, as already demonstrated for the PI3K class II [65].

### 3.2. Trafficking

The regulation of the pathways involved in KCa3.1 channel trafficking has been proposed as a new strategy for regulating the KCa3.1 current, since the inhibition of endocytosis by the ubiquitin-activating enzyme E1 strongly increases the number of KCa3.1 channels in the membrane [66]. In expression systems, the KCa3.1 channels at the plasma membrane have a relatively short life, being internalized within 60–90 min [67] and targeted for lysosomal degradation [68]. This process requires components of the ESCRT machinery and the small-molecular-weight guanine nucleotide-binding protein Rab7 [68]. Polyubiquitylation mediates the targeting of membrane-residing KCa3.1 channels to the lysosomes, while USP8 regulates the rate of KCa3.1 channel degradation by deubiquitylating KCa3.1 channels prior to lysosomal delivery [69]. This modulation could explain the increase of KCa3.1 current observed following short exposure (90 min) of glioblastoma cells to CXCL12, since noise analysis indicates that the KCa3.1 current increase is due to an increased number of channels in the membrane (our unpublished data), while no changes in the KCa3.1 channel mRNA levels are observed [70].

### 3.3. Transcriptional Regulation

Two main transcription factors have been found to regulate the KCa3.1 channel expression, AP-1 and REST. AP-1 was first identified in T lymphocytes where its activity, stimulated by the ERK1/2 pathway, promotes an increase in KCa3.1 current and cell proliferation [71]. In the glioblastoma cell line GL-15 the inhibition of ERK1/2 by the MEK inhibitors PD98059 reduces the mRNA levels for the KCa3.1 channels, suggesting that the same modulation described in T lymphocytes is also working in glioblastoma models [32]. This modulation is relevant as the ERK1/2 pathway is deregulated in most glioblastomas, because of the several mutations accumulated during gliomagenesis [72]. The second transcription factor found to modulate the KCa3.1 channel expression is REST (Repressor Element 1-Silencing Transcription factor). The *Kcnn4* gene contains two RE-1 sites whose occupancy by REST represses gene transcription. In vascular smooth muscle cells the downregulation of REST correlates with KCa3.1 channel upregulation and proliferation [73]. Thus, changes in glioblastoma REST levels could explain the ERK-independent *Kcnn4* transcriptional downregulation we found in GL-15 glioblastoma cells with time of culture [32]. REST has in fact been shown to negatively regulate the adult CNS differentiation [74, 75], and KCa3.1 mRNA downregulation was found to be accompanied by the appearance of several differentiation markers [32].

## 4. Expression of KCa3.1 Channels in Glioblastoma and Healthy Tissues

Early evidence for the expression of KCa3.1 channels in glioma cells came from biochemical and electrophysiological studies performed about twenty years ago. In rat C6 glioma cell line it was first observed that Ca^2+^ ionophores induced a rubidium flux sensitive to nanomolar concentration of ChTX but not to IbTX, TEA, and apamin [76, 77]. Patch-clamp experiments in the same cell line confirmed the presence of a K-selective channel having a unitary conductance of 26 pS in symmetrical K and a sensitivity to submicromolar [Ca^2+^]_i_ [77, 78]. This channel could also be activated by several physiological Ca^2+^ agonists, such as endothelin, serotonin, histamine, and bradykinin [23, 79–84]. 

Subsequent work from our laboratory showed that the KCa3.1 channel was also expressed in human glioblastoma cell lines (GL-15 and U251; [32, 85]). Coapplication of the Ca^2+^ ionophore ionomycin with the KCa2/KCa3.1 channel activator EBIO evoked in these cell lines a sustained K current inhibited by ChTX, CTL, and TRAM-34 but not by the KCa2 channel blocker d-TC. Single channel recordings confirmed the presence of a unitary K current with biophysical and pharmacological properties congruent with those reported for the cloned human KCa3.1 channel [32–35, 85]. In accordance, the KCa3.1 channel transcripts could be amplified from both GL-15 and U251 cells [32].

Besides the U251 cell line, the KCa3.1 channel transcripts were also found by Sontheimer's group in D54-MG, another human glioblastoma cell line, as well as in a human glioblastoma biopsy [86]. These authors, however, found neither evidence for a KCa3.1 current in these tissues (probed in whole-cell configuration with a [Ca^2+^]_i_ of 750 nM), nor for the KCa3.1 channel protein (using western blot analysis and commercially anti-KCa3.1 antibody) [86]. 

With regard to this apparent discrepancy on the functional expression of KCa3.1 channels in human glioblastoma cells, a third group recently found a substantial level of KCa3.1 channel transcripts in U87 and U251 cell lines, as well as in a glioblastoma biopsy [87]. Moreover, they found that the same cells displayed a voltage insensitive, Ca^2+^-activated K-selective current blocked by CTL and TRAM-34, indicating that the KCa3.1 channel was expressed in human glioblastoma cells. The expression of the KCa3.1 channel protein in glioblastoma cells was further confirmed by the same group with western blot analysis [88]. These authors tried to explain the discrepancy of their results with those of Sontheimer's group by considering the different experimental conditions used in the whole-cell recordings and the different sensitivity of the antibodies used in the western blot analysis.

The high expression of the KCa3.1 channel in glioblastoma cells could have a major diagnostic and therapeutic relevance, provided that its presence in the brain was restricted to the transformed glial cells. Early work performed soon after the cloning of the human KCa3.1 channel showed that the KCa3.1 channel transcripts were not expressed in the human central nervous system, although they were found in many other human tissues (placenta, lung, salivary gland, colon, prostate, thymus, spleen, bone marrow, lymph nodes, lymphocytes, and in many of these tissues the functional expression of the KCa3.1 channel was confirmed by patch clamp experiments) [33–35]. This was confirmed by an RT-PCR study showing that KCa3.1 channel transcripts could be found in D54-MG and U251 human glioblastoma cell lines, as well as in a human glioblastoma biopsy but not in a grade III astrocytoma nor in normal human brain and in cultured rat astrocytes [86]. All these studies strongly suggested that the KCa3.1 channel was only scantly expressed in human normal brain tissue, while being strongly upregulated in glioblastomas.

Data from nonhuman specimen appear instead less clear. Earlier electrophysiological studies focused on normal rat and mouse glial cells did not find any evidence for the expression of the KCa3.1 channel, while reporting the presence of other Ca^2+^-activated K channels such as KCa1 and apamin-sensitive SK channels [87, 89, 90]. The expression of KCa3.1 channels was instead reported in cultured rat microglia [91, 92], but these cells did not appear to express KCa3.1 channels in *in vivo* slices [93]. Currents that could be ascribed to the KCa3.1 channel were observed in rat dorsal root ganglion and autonomous neurons [94–96], and most recently in rat cerebellar Purkinje cells [97]. Immunohistochemical analysis revealed the KCa3.1 channel protein in rat ependymal cells [98]. More recent studies indicate, however, that normal mouse astrocytes express low levels of KCa3.1 channels. More specifically, one study shows that about 10% of GFAP-positive mouse astrocytes is immunoreactive to antibody against KCa3.1 channels, and this percentage increases 5-fold following spinal cord injury. This latter result is consistent with the observation that KCa3.1 channels are highly expressed in activated astrocytes [93]. A second study also reports KCa3.1 immunoreactivity in mouse astrocytes (mostly at the endfoot) and shows that the channel participates to the neurovascular coupling. The study further shows that 50% of GFAP-positive astrocytes in slice preparation expresses TRAM-34 sensitive and NS309-activated KCa3.1 currents [99]. Taken together, these data would suggest that KCa3.1 channels are present in a fraction of normal mouse astrocytes. Further dedicated experiments are needed to conclusively clarify whether human normal astrocytes express KCa3.1 channels, and whether interspecies differences exist in the expression of KCa3.1 channels in the brain.

## 5. Functional Roles of KCa3.1 Channels in Glioblastoma Cells

### 5.1. Cell Proliferation and Growth

KCa3.1 channel expression has been shown to be upregulated in many cancer cell types, and in most of them a role of this channel in promoting cell growth and cell cycle progression has been evidenced (reviewed in [25]). A similar role in glioblastoma cells is suggested by data showing that CTL inhibits the growth of glioblastoma cell lines (by inducing a cell cycle arrest at G1-S transition) and delays the development of intracranial glioblastoma tumor formation [100–102]. However, given the several unspecific effects of CTL, these data do not conclusively show whether KCa3.1 channels have a role in the growth of glioblastoma cells. A recent work aimed at specifically addressing this issue found that both CTL and the more specific CTL analog TRAM-34 inhibited the growth of U87 and U251 cells, although with IC50s much higher than those needed to inhibit channel activity. By contrast, when inhibition of KCa3.1 current (down to 20%) was attained by RNA interference, no measurable effect was observed on cell growth [88]. Based on these observations the authors concluded that KCa3.1 channel activity is unlikely to have a major role in glioblastoma cell proliferation, and the effects of KCa3.1 channel inhibitors are most likely unspecific. It should be noticed, however, that under the assumption that the effect of KCa3.1 channel on cell growth is mediated by the channel-induced hyperpolarization (that would facilitate Ca^2+^ influx through the membrane), an IC50 for cell growth inhibition higher than that for channel block has to be expected, as documented for many K channel blockers (reviewed in [103]). A role of KCa3.1 channels in glioblastoma cells proliferation cannot thus be excluded based on the available data, and further experiments addressing this point are needed.

### 5.2. Cell Migration and Invasion

More conclusive data assign a role to KCa3.1 channels in glioblastoma cell migration. Cell migration plays a crucial role in the pathophysiology of glioblastomas, and several ion channels have been shown to have a major role in this process (*cf.* Section 1). Given the abundant expression of KCa3.1 channels in glioblastoma cells and the substantial role this channel has in the migration of other cell types [27], we recently verified whether glioblastoma cells require KCa3.1 channel activity to move. More specifically, we asked whether physiological motogens likely surrounding glioblastoma cells *in vivo* use KCa3.1 channels for their promigratory activity. Among them, the chemokine CXCL12/SDF-1 appeared of interest as its receptors CXCR4 are widely expressed in glioblastoma tissue [104–107], and their activation plays a key role in the migration of glioblastoma cells [108–110]. Interestingly, we found that KCa3.1 channel activity was required in the chemotactic response to SDF-1 of GL-15 and U251 cell lines, primary cultures and freshly dissociated tissue [70]. The chemotactic response, probed with standard transwell chamber, was indeed strongly attenuated both in presence of TRAM-34 and by KCa3.1 channel silencing by RNA interference. In patch-clamp experiments we found that in a fraction of GL-15 cells brief applications of SDF-1 activate KCa3.1 channels by increasing the intracellular [Ca^2+^]_i_. More prolonged SDF-1 applications (three hours incubation) on GL-15 cells induced instead an upregulation of the maximal KCa3.1 channel conductance, suggesting a posttranslational upregulation of the channel protein.

We further found that the KCa3.1 channel activation is not a general requirement for motogen-induced migration in glioblastoma cells. KCa3.1 channel inhibitors were in fact ineffective in modulating the chemotactic response to epidermal growth factor (EGF), another physiologically relevant chemotactic inducer in glioblastoma [111]. Patch-clamp experiments on GL-15 cells showed that EGF activates a KCa3.1 current very similar to that seen in response to SDF-1. Additional experiments showed that EGF, unlike SDF-1, was not able to upregulate the KCa3.1 channel functional expression following prolonged incubation, suggesting this SDF-1-induced modulation may be the relevant one for chemotaxis.

 Other *in vivo* promigratory signals for glioblastoma cells could be present in the serum that can infiltrate into the tumor area of glioblastomas as result of the blood-brain barrier breakdown [112, 113]. Several studies show that fetal calf serum (FCS) enhances the migration of glioblastoma cells by inducing oscillations of the [Ca^2+^]_i_. [Ca^2+^]_i_ oscillations are thought to facilitate the detachment of focal adhesions, through stimulation of focal adhesion kinase, and the retraction of the cell rear towards the direction of movement [21]. However, since the FCS-induced [Ca^2+^]_i_ oscillations reach peaks sufficiently high to activate KCa3.1 channels, we hypothesized that K efflux through KCa3.1 channels could serve for the volume changes needed during cell migration. We found that in about 40% of U-87 cells, acute application of 10% FCS resulted in an oscillatory activity of a K-selective, TRAM-34 sensitive current, displaying frequencies well within those observed for the FCS-induced [Ca^2+^]_i_ oscillations [114]. Beside inducing a cyclical activation of KCa3.1 channels, FCS also promoted the stable (nonoscillatory) activation of a Cl-selective current having biophysical and pharmacological properties resembling those found for the volume-activated Cl current (ICl, swell) widely expressed in glioblastoma cells. Coherently, transwell migration assays performed in the presence of KCa3.1 and Cl channel inhibitors indicated that the activity of these two channels was needed for the promigratory activity of FCS [114]. Finally, the Cl channel blocker 5-nitro-2-(3-phenylpropil) benzoic acid (NPPB) has been shown to block KCa3.1 channels at concentrations often used to block Cl channels [85], suggesting that the particularly high efficacy of this compound on glioblastoma cell migration [115] is due to its inhibitory effects on both channel types.

### 5.3. Mechanistic Roles of KCa3.1 Channels in Cell Migration

**Figure 1:**
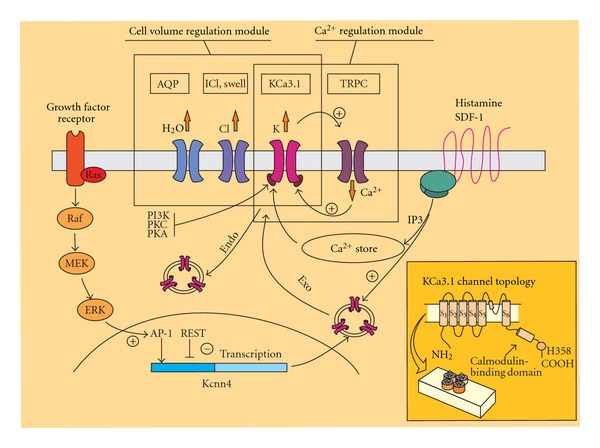
Schematic drawing illustrating the main properties and roles of the KCa3.1 channel expressed in glioblastoma cells. KCa3.1 channels can be activated by elevations of the [Ca^2+^]_i_ originating either from the PLC- and IP_3_-dependent Ca^2+^ release from intracellular stores triggered by G-protein-coupled receptors or from Ca^2+^ influx through TRPC channels. The KCa3.1 channel activity can also be regulated by several kinases, such as PI3K, PKC, and PKA. The expression of the channel is under the control of the RTK/ERK/MAPK-dependent AP-1 and REST transcription factors acting on the *Kcnn4* gene and further depends on the balance between endo- and exocytosis of KCa3.1 channel-containing vesicles. The drawing further highlights the two basic mechanisms sustained by the KCa3.1 channels: (i) inserted in the Ca^2+^ regulation module, in synergy with Ca^2+^ permeable channels (a TRPC in the scheme), the KCa3.1 channel amplifies the Ca^2+^ signals by hyperpolarizing the membrane, thus increasing the driving force for Ca^2+^ influx; (ii) in the cell volume regulation module, in synergy with Cl and aquaporin channels, the KCa3.1 channel controls the cell volume by contributing to changes in the intracellular osmolarity and water content. Inset: Top: KCa3.1 subunit topology showing the six transmembrane domain signature. The calmodulin-binding domain and the histidine phosphorilation site at the C-terminus have been indicated. Bottom: Schematic drawing showing the homotetrameric nature of functional KCa3.1 channels.

As discussed in the Introduction and illustrated in Figure 1, there are two possible mechanisms through which KCa3.1 channels could subserve glioblastoma cell migration. The first mode holds that the channel is instrumental, together with the Cl channel and aquaporins, to the combined outward ion flux needed for cell volume decrease. At relatively low [Ca^2+^]_i_, shown to correspond to the lamellipodium protrusion, the membrane conductance is dominated by the ICl, swell, and the membrane potential is very close to the Cl equilibrium potential (ECl). Under these conditions no transmembrane ion flux through the KCa3.1 and Cl channels is present, since there is no driving force for Cl ions, and KCa3.1 channels are closed. During this period the membrane transporters, usually located at the front of migrating cells [27] will bring ions and water inside the cell, thus allowing the cell volume expansion needed for cell protrusion. By contrast, the opening of KCa3.1 channels during the peaks of [Ca^2+^]_i_ oscillations will move the resting membrane potential to values between EK and ECl, a condition promoting both K and Cl efflux, followed by water for osmotic requirements. The resulting reduction in cell volume, accompanied by the detachment of focal adhesions located at the cell rear [21], would thus facilitate the retraction of the cell body.

Besides controlling cell volume KCa3.1 channels could promote glioblastoma cell migration through the modulation of [Ca^2+^]_i_ signals. Several works have indeed shown that the activity of KCa3.1 channels facilitates the entry of Ca^2+^ ions from the extracellular medium by providing a counter ion to limit cell depolarization and also by hyperpolarizing the cell membrane and increasing the driving force for Ca^2+^ influx. This was first demonstrated in activated T lymphocytes [115] and subsequent works confirmed this role in other cell types expressing this channel [116–118]. In GL-15 cells we found that prolonged applications of histamine induced an increase of [Ca^2+^]_i_ consisting of a fast peak caused by the release of Ca^2+^ from the intracellular stores, followed by a sustained phase dependent on Ca^2+^ influx through a lanthanium-sensitive pathway. Interestingly, the activation of KCa3.1 channels significantly enhanced the sustained phase, as indicated by a reduction of the histamine-induced [Ca^2+^]_i_ in the presence of TRAM-34 [119]. This result strongly suggests that the activation of KCa3.1 channels could contribute to glioblastoma cell migration by modulating the shape of [Ca^2+^]_i_ oscillations. In accordance with this hypothesis, we recently built a theoretical model of [Ca^2+^]_i_ oscillations incorporating the dynamics of the membrane potential and found that a channel activity with the properties of KCa3.1 channels could sensibly affect IP3 driven [Ca^2+^]_i_ oscillations (it increased both the amplitude and duration of each [Ca^2+^]_i_ spike and the oscillatory frequency) [118]. Interestingly, we found that under particular conditions the presence of KCa3.1 channel activity is necessary in order for the cell to generate [Ca^2+^]_i_ oscillations [120, 121]. This last result would explain old experiments showing that the KCa3.1 channel inhibitor ChTX is able to abolish the bradykinin induced [Ca^2+^]_i_ oscillations in C6 glioma cells [23]. Which of the two mechanisms (cell volume regulation or control of the Ca^2+^ influx) is the prominent one in the control of glioblastoma cell migration by KCa3.1 channels remains to be established.

## 6. Concluding Remarks

The data presented here indicate that KCa3.1 channels play a relevant role in cell migration, a critical process in glioblastomas where the spreading and infiltration of their cells into the normal brain parenchyma represent major causes for tumor progression and recurrence following tumor surgical resection. They show in addition that KCa3.1 channels are abundantly expressed in glioblastoma cells, whereas they are only scantly present in healthy human brain tissues. These results combined would point to the KCa3.1 channels as a potential target for newer therapeutic approaches against glioblastomas. KCa3.1 channel blockers are indeed beginning to be considered in therapy, and certain results appear encouraging. First, the KCa3.1 channel blocker TRAM-34, as well as more recently developed analogs have been found to effectively penetrate into the brain and reach interesting brain concentrations after intraperitoneal injection [40, 53]. Second, a KCa3.1 channel inhibitor, Senicapoc from Icagen Inc., has already been used in phase II clinical trials for sickle cell disease and asthma and appears to be well tolerated and safe in humans [26]. Thus this compound could be a convenient starting point to develop effective drugs against glioblastoma. It would be most interesting to investigate whether KCa3.1 channels are expressed in glioblastoma stem cells, and whether they underlie, as in the ordinary glioblastoma cells, the main processes of cell growth, migration, and angiogenesis. This information would also contribute robustly to the comprehension of the glioblastoma pathophysiology. Much remains to be done instead to clarify the diagnostic and prognostic relevance associated with the expression of the KCa3.1 channel in glioblastoma cells. It would be important to this respect to verify whether the level of KCa3.1 channel expression is correlated with the grade of the tumor and the expression of other recognized tumor markers.

 It would also be very important to conclusively clarify the involvement of KCa3.1 channels in the cell cycle progression of glioblastoma cells, and whether their activity is needed for other functional roles relevant to this pathology. Notably, we have preliminary evidence for an effect of TRAM-34 in the glioblastoma-induced angiogenesis, a process that allows glioblastoma cells to ensure themselves for the necessary oxygen and nutrients [122, 123]. The relevance of this study is underpinned by the observation that antiangiogenic therapies are considered clinically very effective and promising [124]. In the hypothesis that a role of KCa3.1 channels in the glioblastoma-induced angiogenesis will be confirmed, the use of KCa3.1 channel inhibitors may be expected particularly effective in the treatment of this pathology, given their inhibitory action on two distinct vital functions for the tumor mass, namely, cell spreading and angiogenesis.
